# Poor retention and care-related sex disparities among youth living with HIV in rural Mozambique

**DOI:** 10.1371/journal.pone.0250921

**Published:** 2021-05-21

**Authors:** Aima A. Ahonkhai, Muktar H. Aliyu, Carolyn M. Audet, Magdalena Bravo, Melynda Simmons, Gael Claquin, Peter Memiah, Anibal N. Fernando, James G. Carlucci, Bryan E. Shepherd, Sara Van Rompaey, Zhihong Yu, Wu Gong, Sten H. Vermund, C. William Wester

**Affiliations:** 1 Division of Infectious Diseases, Vanderbilt University Medical Center, Nashville, Tennessee, United States of America; 2 Vanderbilt Institute for Global Health, Vanderbilt University Medical Center, Nashville, Tennessee, United States of America; 3 Department of Health Policy, Vanderbilt University Medical Center, Nashville, Tennessee, United States of America; 4 Friends in Global Health (FGH), Maputo, Mozambique; 5 MRC/Wits Rural Public Health and Health Transitions Research Unit (Agincourt), School of Public Health, Faculty of Health Sciences, University of the Witwatersrand, Johannesburg, South Africa; 6 Division of Epidemiology and Prevention, University of Maryland School of Medicine, Baltimore, Maryland, United States of America; 7 Provincial Health Directorate of Zambézia, Quelimane, Mozambique; 8 Division of Pediatric Infectious Diseases, Vanderbilt University Medical Center, Nashville, Tennessee, United States of America; 9 Department of Biostatistics, Vanderbilt University School of Medicine, Nashville, Tennessee, United States of America; 10 Yale School of Public Health, Yale University, New Haven, Connecticut, United States of America; Fred Hutchinson Cancer Research Center, UNITED STATES

## Abstract

**Background:**

There are few studies that characterize sex-related differences in HIV outcomes among adolescents and young adults (AYA) 15–24 years of age.

**Methods:**

We conducted a retrospective cohort study among AYA who enrolled in a comprehensive HIV program in Mozambique between 2012–2016. We assessed patients by sex and pregnancy/lactation status, comparing time to combination antiretroviral therapy (ART) initiation using Cox proportional hazard models. We employed multivariable logistic regression to investigate pre- and post-ART retention. Patients were defined as ‘retained pre-ART’ if they attended at least 3 of 4 required visits or started ART in the 6 months after enrollment, and ‘retained post-ART’ if they had any ART pickup or clinical visit during the last 90 days of the one-year follow-up period.

**Results:**

Of 47,702 patients in the cohort, 81% (n = 38,511) were female and 19% (n = 9,191) were male. Of the females, 57% (n = 21,770) were non-pregnant and non-lactating (NPNL) and 43% (n = 16,741) were pregnant or lactating (PL). PL (aHR 2.64, 95%CI:2.47–2.81) and NPNL females (aHR 1.36, 95%CI:1.30–1.42) were more likely to initiate ART than males. PL females had higher odds of pre-ART retention in care (aOR 3.56, 95%CI: 3.30–3.84), as did NPNL females (aOR 1.71, 95%CI: 1.62–1.81), compared to males. This was also true for retention post-ART initiation, with higher odds for both PL (aOR 1.78, 95%CI:1.63–1.94) and NPNL females (aOR 1.50, 95%CI:1.35–1.65) compared to males.

**Conclusions:**

PL females were most likely to initiate ART and remain in care post-ART in this AYA cohort, likely reflecting expansion of Option B+. Despite pregnancy and policy driven factors, we observed important sex-related disparities in this cohort. NPNL females were more likely to initiate ART and be retained in care before and after ART initiation than males. These data suggest that young males need targeted interventions to improve these important care continuum outcomes.

## Introduction

Combination antiretroviral therapy (ART) scale-up has led to unprecedented progress in the global HIV/AIDS response, but these gains have not fully benefited the four million adolescents and young adults (15–24 years of age) living with HIV (AYA-HIV) [1]. Eighty-five percent of AYA reside in sub-Saharan Africa (SSA); and half of the 15–19 year-olds living with HIV reside in just six countries—Mozambique, South Africa, Nigeria, Kenya, India, and Tanzania [1–4]. Mozambique is a resource-constrained nation with high fertility and mortality rates, and an adult HIV prevalence of 13.2% [5]. Mozambique’s population is predominantly young, as 66% of the population is less than 25 years of age, and approximately 7% of the 2.2 million people living with HIV in Mozambique are adolescents and young adults (AYA) [6, 7]. AYA have increasingly been recognized as an important key sub-population for HIV outcomes [8]. This is especially true in SSA, where the HIV prevalence among young people is 6.9%, with relatively higher prevalence among young women (9.8%) or youth between 23–24 years of age (14.9%) [5]. HIV is also now the leading cause of death in this age group [7].

Adolescence and young adulthood are typically considered a period of optimal physical health, but this time is also a vulnerable developmental period for health outcomes [3]. Adolescence is characterized by impulsivity, risk-taking, concrete thinking, and variable levels of social support and autonomy. These developmental features may create obstacles for effective adherence to chronic medical therapies [8–10]. It is no surprise then, that AYA-HIV have poor adherence to ART and reduced utilization of HIV services [8, 11, 10]. Whereas approximately 25% of youth may default from care, one-third to one-half of AYA who remain in HIV care develop virologic failure or viral rebound after initial suppression due to poor adherence [8, 9, 11, 12].

In addition to the unique developmental features of adolescence, sex disparities play an important role in the adolescent HIV crisis. While some data suggest important sex-related differences in HIV viral load and CD4 percentage, the impact on long-term outcomes is unclear [13, 14]. In addition, complex social factors contribute to the risk of HIV acquisition among adolescent girls and young women in SSA [15]. Key among them are gender norms that negatively impact educational and socioeconomic opportunities for adolescent girls and young women [15]. In Maputo Province in Mozambique, for example, 25% of females 20–24 years of age were married before age 15, and 40% had given birth before age 18 [16]. Young women also acquire HIV 5–7 years earlier than men, likely due to earlier sexual debut and partnerships with older men [6]. Partly as a result of these factors, in Mozambique the HIV prevalence among females (15.4%) is significantly higher than among males (10.1%) [5].

AYA-HIV who are pregnant or breastfeeding are an especially vulnerable group [17]. Despite the importance of HIV care for the prevention of mother-to child transmission (PMTCT), high rates of attrition have been reported among pregnant women in SSA [17, 18]. A systematic review of the PMTCT cascade found that 49% of pregnant women were lost to follow-up (LTFU) prior to delivery, and 34% were LTFU within 3 months of delivery [19]. Another study from rural Mozambique found that more than half of women who were pregnant or lactating (median age 24 years) were LTFU; and had a 2- to 3-fold increased risk of LTFU from HIV care than women who were non-pregnant and non-lactating [20]. Even cohorts reporting better estimates of retention report that attrition is highest soon after ART initiation, and among women initiating ART under Option B+ [20–22].

With the higher prevalence and earlier disease acquisition described among adolescent girls and young women, much of the focus for study and intervention has been on this high-risk group [7, 16, 23]. In contrast, adolescent boys and young men have received less attention. The limited data that are available suggest that male AYA have worse outcomes than female AYA across the care continuum, including higher mortality [1, 24]. The objectives of this analysis were to summarize performance at key steps along the HIV care continuum, including time to ART initiation, pre- and post-ART retention, and 1-year mortality to identify sex-related disparities for AYA living with HIV in a large HIV program in Mozambique.

## Materials and methods

### Setting

Friends in Global Health (FGH) is a not-for-profit wholly owned subsidiary of Vanderbilt University Medical Center (a Vanderbilt Health Services entity) that has been registered with the Mozambican government since 2006. Funded by the U.S. Centers for Disease Control and Prevention (CDC) / the U.S. President’s Emergency Plan for AIDS Relief (PEPFAR), FGH supports the sustainable implementation of Ministry of Health (MOH) HIV and TB services in Zambézia Province. During the time of this analysis, FGH was supporting 89 health facilities within 10 districts in Zambézia Province with more than 80,000 persons receiving ART as of July 2019.

### Study design

We conducted a retrospective cohort study of AYA 15–24 years of age who enrolled in the HIV program between September 30, 2012 and January 20, 2017 (pre-ART retention analysis) and between September 30, 2012 and September 20, 2016 (post-ART retention analysis) in 89 FGH-supported health facilities. Patients were categorized by sex and pregnancy/lactation status at enrollment (i.e. male, female pregnant or lactating [PL], female non-pregnant and non-lactating [NPNL]). Data were censored and retention outcomes assessed on September 20, 2017 to permit at least 12 months follow-up for the post-ART retention analysis, and 8 months of follow-up for the pre-ART retention analysis. All data were collected from the OpenMRS database, which included patient demographic information, visit dates, ART refill information, and laboratory values. While patients were naïve to care at their enrollment site, documentation of transfer status was not available in the OpenMRS database. Patients who were ART-experienced at enrollment were not included in this analysis.

### Outcome measures

We identified four outcomes representing key clinical and process outcomes along the continuum of HIV care: 1) **Time to ART initiation** was measured from the date of participant enrollment in the clinic to the date of first ART pick-up, 2) **Pre-ART retention** was measured at 6 months. The typical visit schedule consists of 4 visits: at time zero (enrollment), 1 month, 3 months, and 6 months. Patients who attended 2 out of 3 follow-up visits, and/or initiated ART within 6 months of enrollment (including PL females who initiated ART under Option B+) were defined as being retained in care. Patients who had documented transfer of care within 6 months of enrollment were assumed to be retained. Conversely, those who died during this period were considered as not retained in care, 3) **Post-ART retention** was measured at the end of the 12-month follow-up period. Patients were classified as retained in care if they had any least one ART pick-up or clinical visit in the last 3 months of the follow-up period. Patients with documented transfers of care to another facility were deemed to be retained in care. 4) Observed one-year **mortality** was defined as death within 12 months of ART initiation.

### Statistical analysis

#### Baseline demographic and clinical parameters

We summarized baseline individual, health facility, and community factors. Individual factors were sex, pregnancy/lactation status (NPNL vs. PL females), age category (adolescents 15–19 years of age vs. young adults 20–24 years of age), educational level (none vs. primary vs. secondary or higher education), marital status (married vs. living with partner vs. single), baseline WHO clinical stage, year of enrollment into care, and ART treatment policy at enrollment. We considered 3 treatment policy periods: 1) the policy to initiate ART at CD4 cell count <350 cells/mm^3^ spanned from September 30, 2012 to February 29, 2016; 2) the policy to initiate ART at CD4 cell count <500 cells/mm^3^ spanned from March 1, 2016 to April 20 2017; and, 3) Test-and-Start (ART commenced and continued lifelong, regardless of CD4 cell count) was rolled out between March 1, 2016 and April 11, 2017 depending on the district, and continued through the censor date. Option B+ was operationalized in Mozambique in June of 2013, but this was not considered a separate ART treatment policy, since it only applies to PL females who were accounted for separately in our analyses. Health facility factors included the service delivery point at which individuals were initially diagnosed with HIV and referred into care including maternal and child health (MCH) clinics, free standing voluntary counseling and testing (VCT) centers, provider-initiated testing and counseling (PITC) services, and others. We also summarized the data by the setting of the healthcare facility including district capital (i.e. main health facility for the district), district peripheral, urban large (i.e. health facility with ≥ 2,000 active patients on ART), or urban other (i.e. health facility with < 2,000 active patients on ART). Descriptive statistics included frequency, percentage, mean, standard deviation, median, interquartile range (IQR). P-values were computed for categorical and ordinal variables using a chi-squared test with Yates continuity correction.

#### Pre-and post-ART retention

We used multivariable logistic regression models to determine the odds of pre- and post-ART retention among individuals by sex and pregnancy/lactation status. Models were adjusted for individual and health system factors described above, adjusting for covariates known to be associated with retention, and/or potential confounders of the relationship between sex and pregnancy/lactation status and retention. Two-sided 95% Wald confidence intervals are provided. We also assessed whether the relationship between sex and pregnancy/lactation status and retention differed by age category by fitting an interaction term. Non-available data were treated as missing values. To account for the missing data, we performed multiple imputation and pooled results from 25 imputed datasets. Results were similar when non-available data were treated as a subcategory in analyses (S1 Fig).

#### Time to ART initiation

We used cumulative incidence plots, accounting for death as a competing risk, to illustrate the probability of ART initiation from the time of enrollment for NPNL females, PL females, and males. We used multivariable Cox proportional hazards models to compare the chance (‘hazard’) of ART initiation among NPNL females, PL females, and males. Models were adjusted for individual and health system factors described above. Again, we assessed whether the relationship between sex and pregnancy/lactation status and retention differed by age category by fitting an interaction term.

#### Mortality

Observed one-year mortality was defined as death within 12 months following ART initiation. Pre-ART mortality was not included in this estimate. We used a published nomogram, which estimates the proportion of deaths among those classified as lost to follow up (LTFU), to calculate nomogram-adjusted mortality in the entire cohort according the formula below:

$$M_{C}=(1-r)M_{NL}+rM_{L}$$

where **M**_**c**_ = nomogram adjusted mortality, **r** = Proportion lost to follow-up, **M**_**NL**_ = Mortality observed in patients retained in care (not lost to follow-up), **M**_**L**_ = Mortality estimated in patients lost to follow-up [25]. **M**_**NL**_ was calculated directly from our dataset. LTFU was defined as no ART pickup or clinical visit within 60 days of the expected ART pickup date. Mortality in the LTFU group (***M***_***L***_) was estimated from a meta-regression analysis of 15 studies focused on ART-treated patients in SSA [26]. Studies included in the meta-analysis reported outcomes among LTFU who underwent contact tracing to ascertain vital status. These data showed an inverse relationship between mortality in the LTFU group and the rate of loss to follow-up in the program [27].

All analyses were performed using R version 3.5.2 [28].

#### Ethics statement

An electronic Open Medical Record System (OpenMRS)^™^ is utilized at VUMC/FGH-supported health facilities to facilitate patient care and program monitoring and evaluation activities [29]. De-identified data were extracted from the OpenMRS database for this secondary analysis. The data use and evaluation plan, with a waiver of consent, for this study were approved by the Committee of Bioethics in Health in Zambézia, Mozambique (Comité Institucional de Bioética para Saúde—Zambézia, Ref: 02/CIBS-Z/16), the Vanderbilt University Medical Center (VUMC) Institutional Review Board (#170970 and #160549), and the (funding agency) Centers for Disease Control and Prevention (CDC) (2016-163/163a).

## Results

### Baseline demographics

**Fig 1:**
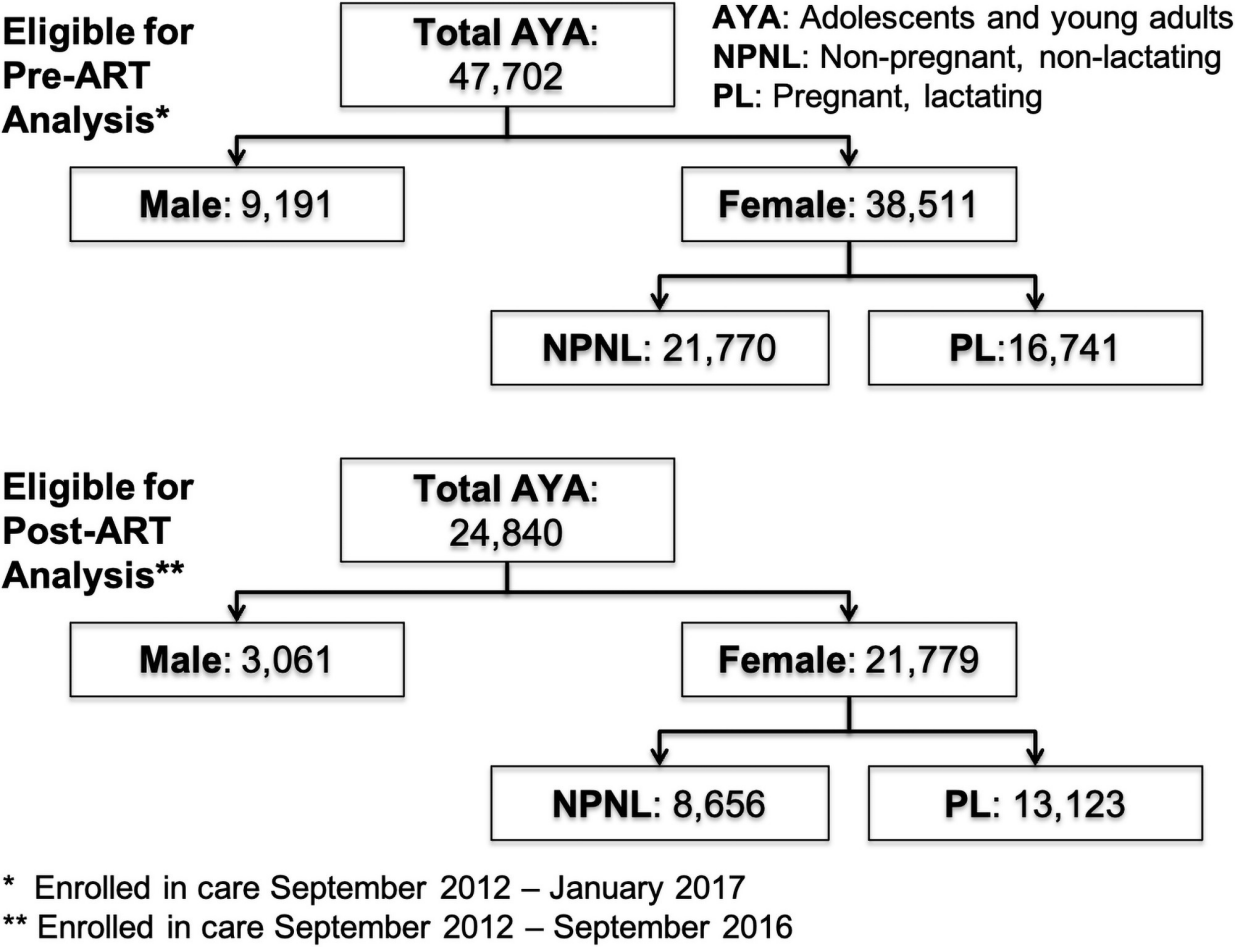
Flow diagram of eligibility for pre- and post- antiretroviral viral therapy analysis.

The cohort consisted of 47,702 AYA who enrolled in care between September 30, 2012 and June 20, 2017. Of these, 46% (n = 21,770) were NPNL females, 35% (n = 16,741) were PL females, and 19% (n = 9,191) were males [Fig 1]. Adolescents (15–19 years of age) comprised 35% (n = 16,763) and young adults (20–24 years of age) comprised 65% (n = 30,939) of the cohort. Twelve percent of patients (n = 5,865) had no formal education. More patients reported living with a partner (33%, n = 15,783), than being single and not cohabitating (26%, n = 12,296), or being married (14%, n = 6,616). The majority of patients (77%, n = 36,630) enrolled in care when guidelines stipulated that ART should be initiated for CD4 cell counts <350 cells/mm^3^; 20% (n = 9,477) enrolled in care when the guidelines stipulated starting ART for CD4 cell count <500 cells/mm^3^, and 3% (n = 1,495) enrolled in care during the Test-and-Start era. Significantly lower proportions of PL females (1%, n = 492) presented with advanced clinical disease (WHO clinical stage 3/4) than NPNL females (11%, n = 5,015) or males (5%, n = 2,413). More patients referred into care from MCH facilities (36%, n = 17,005) than from VCT services (33%, n = 15,640), PITC (15%, n = 6,920), or other health facilities (17%; 8,137) [Table 1].

**Table 1:** Baseline cohort characteristics.

Variable	Category	Total	Male		Female				p-value
					Non-Pregnant and Non-Lactating		Pregnant or Lactating		
		N	n	%	n	%	n	%	
		47,702	9,191	19%	21,770	46%	16,741	35%	
**Policy**	CD4 cell count < 350 cells/mm^3^	36,730	6,847	19%	17,210	47%	12,673	34%	<0.001
	CD4 cell count < 500cells/mm^3^	9,477	2,084	22%	3,873	41%	3,520	37%	
	Test-and-Start	1,495	260	17%	687	46%	548	37%	
**Year**	2013	8,219	1,485	18%	4,267	52%	2,467	30%	<0.001
	2014	11,187	1,861	17%	5,272	47%	4,054	36%	
	2015	12,156	2,396	20%	5,433	45%	4,327	35%	
	2016	11,952	2,540	21%	5,076	43%	4,336	36%	
	2017	4,188	909	22%	1,722	41%	1,557	37%	
**Age Group**	15–19 Years of age	16,763	1,849	11%	7,617	45%	7,297	44%	<0.001
	20–24 Years of age	30,939	7,342	24%	14,153	46%	9,444	30%	
**Education**	No Formal Education	5,865	633	11%	2883	49%	2,349	40%	<0.001
	Primary	21,718	4,096	19%	9,821	45%	7,801	36%	
	Secondary or More	9,669	2,767	29%	3,892	40%	3,010	31%	
	Missing Data	10,450	1,695	16%	5,174	50%	3,581	34%	
**Marital Status**	Married	6,616	1,045	16%	2,212	33%	3,359	51%	<0.001
	Living with Partner	15,783	3,100	20%	6,196	39%	6,487	41%	
	Single	12,296	2,634	22%	7,280	59%	2,382	19%	
	Missing Data	13,007	2,412	18%	6,082	47%	4,513	35%	
**BMI Category**	Underweight	8,271	1,771	21%	5,127	62%	1,373	17%	<0.001
	Normal	25,234	5,048	20%	10,420	41%	9,766	39%	
	Overweight	3,044	241	8%	1,068	35%	1,735	57%	
	Missing Data	11,153	2,131	19%	5,155	46%	3,867	35%	
**CD4 cell count (cells/mm**^**3**^**)**	<100	2,146	692	32%	1,252	59%	202	9%	<0.001
	100–199	2,522	676	27%	1,368	54%	478	19%	
	200–349	5,487	1,270	23%	2,476	45%	1,741	32%	
	350–499	5,957	1,215	20%	2,560	43%	2,182	37%	
	> = 500	10,801	1,659	15%	4,972	46%	4,170	39%	
	Missing Data	20,789	3,679	18%	9,142	44%	7,968	38%	
**WHO Clinical Stage**	Stage 1	28,960	4,088	14%	10,570	37%	14,302	49%	<0.001
	Stage 2	7,923	1,831	23%	4,643	59%	1,449	18%	
	Stage 3	6,963	2,083	30%	4,427	64%	453	6%	
	Stage 4	957	330	35%	588	61%	39	4%	
	Missing Data	2899	859	30%	1,542	53%	498	17%	
**Referral Type**	Any MCH	17,005	1,106	7%	2,557	15%	13,342	78%	<0.001
	VCT	15,640	4,529	29%	10,196	65%	915	6%	
	PITC	6,920	1,656	24%	4,584	66%	680	10%	
	Others	8,137	1,900	23%	4,433	55%	1,804	22%	
**Districts**	Quelimane	14,470	2,597	18%	7,271	50%	4,602	32%	<0.001
	Namacurra	6,196	1,134	18%	2,434	39%	2,628	42%	
	Others	27,036	5,460	20%	12,065	45%	9,511	35%	
**Health Facility Type**	District Periphery	18,632	3,641	20%	8,144	44%	6,847	37%	<0.001
	District Capital	14,600	2,953	20%	6,355	44%	5,292	36%	
	Urban Other	9,044	1,589	18%	4,455	49%	3,000	33%	
	Urban Large	5,426	1,008	19%	2,816	51%	1,602	30%	

MCH: Maternal Child Health VCT: voluntary counselling and testing.

PITC: provider-initiated testing and counseling.

### Pre-ART retention and ART initiation

Overall, 70% of the cohort were retained in care prior to ART initiation [Table 2] and retention improved over the analysis period (compared to 2013; 2014: aOR 2.00 [95%CI 1.88–2.12; 2015: aOR 2.75 [95%CI 2.58–2.92; 2016: aOR 3.58 [95%CI 3.30–3.88]; 2017: aOR 4.02 [95%CI 3.52–4.59]) [Table 3]. Of note, compared to males, PL females had an adjusted OR of 3.56 [95%CI 3.30–3.84] and NPNL females had an adjusted OR of 1.71 [95%CI 1.62–1.81] of being retained in care pre-ART [Table 3]. PL females (aHR 2.64, [95%CI 2.47–2.81]) and NPNL females (aHR 1.36 [95%CI 1.30–1.42)] were more likely to initiate ART than males.

**Table 2:** Pre- and post-ART retention outcomes.

Variable	Category	Total		Male		Female				
						Non-Pregnant and Non-Lactating		Pregnant or Lactating		
				**N = 9.191**		**N = 21,770**		**N = 16,741**		
		**n**	**%**	**n**	**%**	**n**	**%**	**n**	**%**	**p-value**
**Pre-ART Retention** (N = 47,702)	No	14,356	30%	3,813	41%	7,255	33%	3,288	20%	<0.001
	Yes	33,346	70%	5,378	59%	14,515	66%	13,453	80%	
				**N = 3,061**		**N = 8,656**		**N = 13,123**		
		**n**	**%**	**n**	**%**	**n**	**%**	**n**	**%**	**p-value**
**Post-ART Retention** (N = 24,840)	No	12,661	51%	1,666	54%	3,854	45%	7,139	54%	<0.001
	Yes	12,179	49%	1,395	46%	4,800	55%	5,984	46%	

**Table 3:** Odds of being retained in care before ART initiation^+^.

Variable	Category	OR		aOR	
		OR	95% CI	aOR	95% CI
**Policy**	CD4 cell count < 350 cells/mm^3^	Ref		Ref	
	CD4 cell count < 500 cells/mm^3^	2.12	2.01, 2.24	1.33	1.21, 1.45
	Test-and-Start	3.35	2.88, 3.90	1.38	1.15, 1.66
**Year**	2013	Ref		Ref	
	2014	1.93	1.82, 2.05	2.00	1.88, 2.12
	2015	2.52	2.37, 2.67	2.75	2.58, 2.92
	2016	3.64	3.42, 3.87	3.58	3.30, 3.88
	2017	4.63	4.23, 5.07	4.02	3.52, 4.59
**Sex Group**	Male	Ref		Ref	
	Female Non-Pregnant, Non-Lactating	1.42	1.35, 1.49	1.71	1.62, 1.81
	Female Pregnant, Lactating	2.90	2.74, 3.07	3.56	3.30, 3.84
**Age Group**	15–19 Years of age	Ref		Ref	
	20–24 Years of age	1.05	1.01, 1.10	1.16	1.11, 1.21
**Education**	No Formal Education	Ref		Ref	
	Primary	0.93	0.88, 1.00	1.01	0.95, 1.08
	Secondary or More	1.04	0.96, 1.11	1.14	1.05, 1.23
**Marital Status**	Married	Ref		Ref	
	Living with Partner	0.82	0.77, 0.88	0.98	0.92, 1.05
	Single	0.77	0.72, 0.82	0.97	0.90, 1.04
**BMI Category**	Underweight (BMI<18.5)	Ref		Ref	
	Normal Weight (BMI: 18.5–24.9)	1.10	1.04, 1.17	0.99	0.93, 1.06
	Overweight (BMI: 25–29.9)	1.35	1.23, 1.48	0.98	0.88, 1.09
**CD4 cell count**	<100*	Ref		Ref	
	<200*	1.24	1.11, 1.39	1.19	1.06, 1.34
	<350*	1.23	1.12, 1.35	1.03	0.93, 1.14
	<500*	1.03	0.93, 1.13	0.81	0.73, 0.90
	> = 500*	0.89	0.82, 0.97	0.69	0.62, 0.76
**WHO Clinical Stage**	Stage 1	Ref		Ref	
	Stage 2	0.77	0.73, 0.81	0.98	0.93, 1.04
	Stage 3	0.91	0.86, 0.96	1.31	1.22, 1.40
	Stage 4	0.76	0.66, 0.88	1.21	1.04, 1.41
**Referral Type**	Any Maternal Child Health	Ref		Ref	
	VCT	0.57	0.54, 0.60	0.91	0.85, 0.98
	PITC	0.50	0.47, 0.53	0.76	0.71, 0.82
	Others	0.66	0.62, 0.70	0.94	0.87, 1.01
**Districts**	Others	Ref			
	Namacurra	1.27	1.20, 1.35	NA	NA
	Quelimane	1.34	1.28, 1.40	NA	NA
**Health Facility Type**	District Periphery	Ref		Ref	
	District Capital	0.93	0.89, 0.97	1.10	1.04, 1.15
	Urban Other	1.18	1.12, 1.25	1.41	1.33, 1.51
	Urban Large	1.34	1.25, 1.44	1.64	1.51, 1.77

CI = Confidence Interval; OR = Odds Ratio; Ref = Reference.

^**+**^ The multivariable logistic model was adjusted for all variables except “District” since there was substantial overlap between this variable and health facility type.

In adjusted analysis, young adults were more likely to be retained in care before ART initiation than adolescents (aOR 1.16, 95%CI: of 1.11–1.21) [Table 3]. Notably, there was little evidence of an interaction (p = 0.52) between age category and sex/pregnancy/lactation category pre-ART retention.

### One year post-ART retention

There were 24,840 eligible patients in the post-ART retention analysis. Of these, 49% (n = 12,179) were retained in care at 1-year following ART initiation [Table 2]. The odds of being retained in care was greater at the end of the study period in 2016 relative to the beginning in 2013 (aOR 1.27 [95%CI 1.14–1.41]). Young adults (20–24 years of age) had a higher likelihood of being retained in care post-ART when compared to adolescents (15–19 years of age) (aOR adjusted odds ratio (aOR) = 1.30, [95%CI 1.23–1.37]) [Table 4]. Of note, the post-ART 1-year retention rates were greatest for NPNL females (56%, n = 4,800), and similarly lower for PL females (46%, n = 5,984) and males (46%, n = 1,395). After adjusting for ART guideline policies and other covariates, female AYA were more likely to be retained in care one year following ART initiation irrespective of ART guideline policy (aOR 1.78 [95%CI 1.62–1.94] and aOR 1.50 [95%CI 1.35–1.65] for NPNL and PL females, respectively, compared to males) [Table 4]. Additionally, in both unadjusted (OR 0.81 [95%CI 0.68–0.95]) and adjusted analysis (aOR 0.72 [95%CI 0.60–0.86]), those who were categorized with WHO clinical stage 4 had a lower likelihood of being retained in care post-ART [Table 4]. There was little evidence of an interaction (p = 0.24) between age category and sex/pregnancy/lactation category on post-ART retention.

**Table 4:** Odds of being retained in care one year after ART initiation_+_.

Variable	Category	OR		aOR	
		OR	95% CI	aOR	95% CI
**Policy**	CD4 cell count < 350 cells/mm^3^	Ref		Ref	
	CD4 cell count < 500 cells/mm^3^	1.64	1.53, 1.74	1.31	1.19, 1.44
	Test-and-Start	1.42	1.15, 1.75	1.45	1.15, 1.82
**Year**	2013	Ref		Ref	
	2014	0.78	0.72, 0.85	0.85	0.78, 0.93
	2015	1.01	0.93, 1.10	1.10	1.01, 1.20
	2016	1.37	1.26, 1.49	1.27	1.14, 1.41
**Sex Group**	Male	Ref		Ref	
	Female Non-Pregnant, Non-Lactating	1.49	1.37, 1.61	1.78	1.63, 1.94
	Female Pregnant, Lactating	1.00	0.93, 1.08	1.50	1.35, 1.65
**Age Group**	15–19 Years of age	Ref		Ref	
	20–24 Years of age	1.36	1.29, 1.43	1.30	1.23, 1.37
**Education**	No Formal Education	Ref		Ref	
	Primary	1.10	1.02, 1.19	1.18	1.09, 1.27
	Secondary or More	1.31	1.20, 1.44	1.38	1.26, 1.52
**Marital Status**	Married	Ref		Ref	
	Living with Partner	1.25	1.17, 1.34	1.22	1.13, 1.31
	Single	1.29	1.20, 1.39	1.13	1.04, 1.23
**BMI Category**	Underweight (BMI<18.5)	Ref		Ref	
	Normal Weight (BMI: 18.5–24.9)	1.06	0.99, 1.13	1.17	1.08, 1.26
	Overweight (BMI: 25–29.9)	1.04	0.93, 1.15	1.15	1.02, 1.29
**CD4 cell count**	<100	Ref		Ref	
	<200	1.05	0.90, 1.21	1.05	0.90, 1.22
	<350	1.11	0.98, 1.26	1.15	1.00, 1.31
	<500	0.96	0.85, 1.09	1.00	0.87, 1.14
	> = 500	0.89	0.79, 1.01	1.00	0.87, 1.15
**WHO Clinical Stage**	Stage 1	Ref		Ref	
	Stage 2	1.30	1.21, 1.40	1.13	1.05, 1.22
	Stage 3	1.24	1.16, 1.32	1.10	1.01, 1.19
	Stage 4	0.81	0.68, 0.95	0.72	0.72, 0.86
**District**	Other	Ref		NA	
	Namacurra	0.78	0.72, 0.84	NA	NA
	Quelimane	0.70	0.66, 0.75	NA	NA
**Referral Type**	Any Maternal Child Health	Ref		Ref	
	VCT	1.58	1.48, 1.67	1.41	1.30, 1.53
	PITC	1.22	1.13, 1.33	1.20	1.09, 1.32
	Other	1.20	1.11, 1.29	1.38	1.27, 1.51
**Health Facility Type**	District Periphery	Ref		Ref	
	District Capital	1.58	1.48, 1.67	1.07	1.00, 1.13
	Urban Other	1.22	1.13, 1.33	0.56	0.51, 0.61
	Urban Large	1.20	1.11, 1.29	0.92	0.83, 1.10

CI = Confidence Interval; OR = Odds Ratio; Ref = Reference.

^**+**^ The multivariable logistic model was adjusted for all variables except “District” since there was substantial overlap between this variable and health facility type.

### Mortality rates (1-year post ART initiation)

Among patients included in the post-ART retention analysis (n = 24,840), 1.5% (95%CI 1.3–1.5%, n = 370) died within one year of ART initiation. Crude mortality rates were higher for males (3.3%, [95%CI 2.7–4.1%)] than for females (1.3%, [95%CI 1.1–1.4%]), overall. The percentage of patients who were LTFU was 51% (n = 12,661); 50.5% (n = 10,995) for females and 54.4% (n = 1,666) for males. Mortality estimates corrected for LTFU were 11.4% overall (95% CI 3.2–26.5%) and were very similar between males and females (11.9% [95%CI 3.7%–28%] for males vs. 11.4% [95% CI 3.1%–26.4%] for females respectively) [Table 5].

**Table 5:** Mortality outcomes.

Variable	Total		Male		Female			
					Non-Pregnant and Non-Lactating		Pregnant or Lactating	
	N	%	n	%	n	%	n	%
	(N = 47,702)		(N = 9,191)		(N = 21,770)		(N = 16,741)	
**Crude Mortality**	370	1%	102	3%	268	1%	0	0%
**Nomogram-Adjusted Mortality**		11%		12%	11%			

## Discussion

Our study highlights important sex-related differences in ART initiation and retention in care among AYA living with HIV in Mozambique in an era of expanded ART. Most (81%) of this cohort were female and 35% were pregnant or lactating. PL females in our cohort were more likely to initiate ART and to be retained in care prior to ART initiation than NPNL females or males, despite higher baseline CD4 and lower WHO clinical stages at presentation, which may reflect the roll out and scale up of Option B+. While females overall were more likely to initiate ART and to be retained in care when compared to their male counterparts, some, but not all, of these sex-related differences appeared to be driven by pregnancy and/or lactation status. Importantly, despite these notable sex-related differences and overall improvement in retention over the course of the study period, retention among the subset of AYA who initiated ART remained very poor, with only 49% of those who started ART being retained in care 1-year following ART initiation.

Many cohort studies have identified PL females as a particularly vulnerable group for attrition from HIV care [30, 31]. Unfortunately, these studies also suggest that LTFU in this group has worsened despite increased numbers of pregnant women initiating ART under the Option B+ policy, which promotes lifelong ART for PL females regardless of CD4 count [31]. Points along the PMTCT care continuum where attrition is particularly problematic for PL females include: i) immediately following initial HIV diagnosis, ii) post ART initiation and/or enrollment in PMTCT programs, as well as iii) following delivery [21, 31]. While there has been less focus in the published literature on pregnant adolescents, a study from East Africa showed that pregnant adolescents (15–19 years of age) had a 2.5-fold increased risk of LTFU compared to adolescent males [30]. In our analysis, PL females had the highest rates of retention in HIV care at 1 year, and outcomes among PL females were similar between adolescents and young adults.

Generally, post-ART retention was low for all groups (49%), including PL females (46%), in the year following ART initiation and among those in WHO clinical stage 4. In unadjusted analysis, NPNL females had a 50% higher odds of being retained in care compared to AYA males, while PL females had no difference in risk of LTFU. However, adjusting for the policy period (CD4 cell count threshold < 350 cells/mm^3^ vs. < 500 cells/mm^3^ vs. Test-and-Start), AYA females, irrespective of pregnancy or lactation status were more likely to be retained in care in the first year after ART initiation. Compared to other studies, our finding of improved retention of female AYA following ART initiation is unique. This may be attributed to the MOH and FGH efforts to increase retention in Option B+ through (1) partnering with traditional birth attendants and male peer educators to encourage partners of pregnant women to accompany them to their antenatal care visits and to receive HIV counseling and testing, (2) offering peer support for HIV-positive women during pregnancy and breastfeeding periods, (3) supporting the implementation of PMTCT programs through clinical mentoring and supervision, or (4) especially poor retention of male AYA in our setting [32, 33].

In addition, the fact that our entire cohort is comprised of AYA may distinguish our study from others, that have compared AYA to older adults and/or younger children. Our findings may suggest competing priorities among AYA males for whom retention was the poorest in our analysis and underscores the importance of effectively engaging AYA males in HIV care. Despite accounting for only 20% of the cohort, these AYA males presented to care with more advanced HIV disease, had a longer time to ART initiation, and were less likely to be retained in care, both before and after ART initiation. It has been shown that males, in general, perform poorly across the entire HIV care continuum and have a 50% higher risk of mortality compared to females [34–36]. There are a paucity of studies looking specifically at sex disparities in mortality among African youth. One large, multi-site study from the IeDEA consortium which assessed mortality among adolescents, but not young adults, found a 20% higher mortality risk among young females compared to young males [37]. In contrast, our data showed higher observed mortality among young men compared to young women, which may speak to regional differences in our cohort compared to the larger IeDEA consortium and the expected consequences of the delays in ART initiation seen among AYA males compared to females in our cohort [38].

The sex disparities we observed may be related to differences in health-seeking behavior between men and women [34, 36, 37]. Indeed, women are familiarized with the healthcare system via access of reproductive health services, while men may be socialized to view sickness as a weakness and a threat to traditional notions of masculinity. Additionally, in mobile communities employment migration can marginalize males from local healthcare services.[36, 39] Despite this knowledge, male engagement strategies have tended to be tied to marriage and family partnerships [36, 39]. One notable exception to this generalization may be the scale up of voluntary male circumcision. Nonetheless, there is a dearth of focus on the unique needs and challenges of AYA males living with HIV in resource-limited settings [36, 39].

Our analysis has several important strengths. Our cohort was large and spanned 89 clinical sites in Mozambique, improving its generalizability. Additionally, we were able to distinguish PL females from NPNL females, allowing us to assess the effects of having been pregnant and lactating in addition to the comparison of males and females. Despite these strengths, our study also had important limitations. Deaths in this cohort were likely underestimated, as they were under reported in the OpenMRS system. We accounted for this by adjusting for possible deaths among those lost to follow up using a validated nomogram. However, we recognize that this process provides only an estimate of the truth and is not based on the specific age, geographic, and temporal characteristics of our cohort [25]. Additionally, we did not have access to virologic data to allow us to assess sex-related differences in virologic control or access to data on HIV acquisition including perinatally acquired HIV which is known to be associated with poorer retention in care. Like other observational studies using routinely collected data, some of our variables had high levels of missingness (up to 44%). Our analyses attempted to address this limitation using multiple imputation, which assumes that there are no systematic differences between observed and missing values that cannot be explained by other analysis variables. Although this assumption is often made, it is not guaranteed to hold. Finally, while we tried to account for ART initiation policy in the analysis, differential policies applied to special populations (such as Option B+ among PL females) may have introduced bias, which we have acknowledged in the interpretation of our results.

## Conclusions

Our analysis adds to the literature describing very poor retention in care both before and after ART initiation for AYA. We found that sex-related disparities in retention in this age group were not fully be explained by more efficient care received by pregnant women in the era of Option B+, and that adolescent boys and young men are the least likely to initiate ART or be retained in care., Targeted interventions in this setting are also needed to improve very poor outcomes among adolescent boys and young men.

## Supporting information

S1 Fig Flow diagram of eligibility for pre- and post- antiretroviral viral therapy analysis by policy period. (TIFF)

S1 Dataset (CSV)
