# Supplementary figures and images for: Poor retention and care-related sex disparities among youth living with HIV in rural Mozambique
Source: PLoS One. 2021 May 21;16(5):e0250921. doi: 10.1371/journal.pone.0250921 (PMC8139489; doi:10.1371/journal.pone.0250921)

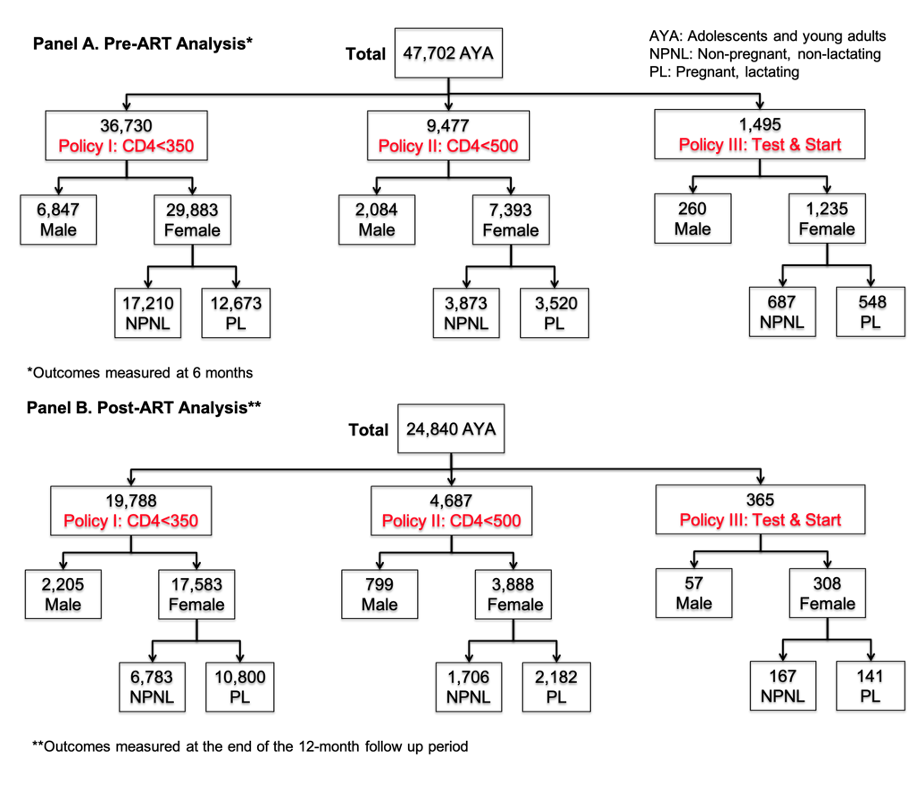

Supplement: S1 Fig — (TIFF) [file pone.0250921.s001.tiff]
